# Opening the Debate: How to Fulfill the Need for Physicians’ Training in Circadian-Related Topics in a Full Medical School Curriculum

**DOI:** 10.5334/jcr.ah

**Published:** 2015-11-05

**Authors:** Julia M Selfridge, Kurtis Moyer, Daniel G S Capelluto, Carla V Finkielstein

**Affiliations:** Virginia Tech Carilion School of Medicine, Roanoke, Virginia; Department of Surgery, Virginia Tech Carilion School of Medicine, Roanoke, Virginia; Department of Biological Sciences, Virginia Bioinformatics Institute, Virginia Tech, Blacksburg, Virginia; Integrated Cellular Responses Laboratory, Department of Biological Sciences, Virginia Bioinformatics Institute, Virginia Tech, Blacksburg, Virginia

**Keywords:** Circadian rhythms, Medical education, Shift work, Occupational health, Circadian disruption, Residency training

## Abstract

**Background:** Circadian rhythms are daily changes in our physiology and behavior that are manifested as patterns of brain wave activity, periodic hormone production, recurring cell regeneration, and other oscillatory biological activities. Their importance to human health is becoming apparent; they are deranged by shift work and jet-lag and in disparate conditions such as insomnia, sleep syndromes, coronary heart attacks, and depression, and are endogenous factors that contribute to cancer development and progression.

**Discussion:** As evidence of the circadian connection to human health has grown, so has the number of Americans experiencing disruption of circadian rhythms due to the demands of an industrialized society. Today, there is a growing work force that experiences night shift work and time-zone shifts shaping the demands on physicians to best meet the needs of patients exposed to chronic circadian disruptions. The diverse range of illness associated with altered rhythms suggests that physicians in various fields will see its impact in their patients. However, medical education, with an already full curriculum, struggles to address this issue.

**Summary:** Here, we emphasize the need for incorporating the topic of circadian rhythms in the medical curriculum and propose strategies to accomplish this goal.

## Background

The dramatist Thomas Dekker wrote in 1611, “*For do but consider what an excellent thing sleep is […] sleep is that golden chain that ties health and our bodies together*” [1]. Four hundred years later, this statement has been repeatedly supported by mounting basic and clinical research and is widely understood to be true. As sleep habits have adapted to changes in technology and occupational demands, Dekker’s words gain new meaning. Accordingly, disruption of sleep patterns alters the timing, quality, and duration of the sleep phase and can be provoked by either a dysfunctional routine that affects the synchronization of a person’s physiology with its external environment or a malfunctioning of the body’s internal timekeeping system. These situations represent new challenges to physicians, who will now need to weigh the influence of a patient’s sleep disruption when determining disease development and progression.

Light-dark cycles synchronize biological functions with the environment in a periodic pattern that takes about 24 h, which is known as the circadian rhythm. Despite growing evidence of the impact that circadian dysfunction has on morbidity and mortality rates for numerous common medical illnesses, topics such as human circadian biology, circadian disorders, and chronobiology have received limited consideration in the medical school curriculum. Today, the few health care professionals who gain adequate competency in these areas do so mostly as a result of postgraduate specialty training. This leaves a large body of professionals unaware of how to *i*) evaluate the clinical relevance of circadian cycle disruption in disease onset, *ii*) optimize therapeutic strategies to include times of the day at which treatment efficacy is most favorable, and *iii*) gauge the relevance of chronotherapies for disease outcome.

As alternative shift work becomes more common in modern societies, the impact of altered night/day shift routines on health is of increasing relevance as it influences a workforce of ~15 million people in the U.S. alone. According to the Bureau of Labor Statistics, in the U.S., approximately 15% of full-time wage and salary employees work evening shifts, night shifts, employer-arranged irregular schedules, or rotating shifts [2]. Disruption of circadian rhythms because of long-term shift work has been implicated in many different illnesses including the top two causes of death among Americans - cardiovascular disease and cancer, most prominently breast and prostate cancers [3,4]. In addition, type 2 diabetes/metabolic syndrome [5], obesity [6], digestive problems [7], and depression [8] are also common consequences of workers’ exposure to chronic shift work [9]. Even short-term health effects (*e.g.*, gastrointestinal symptoms, insomnia, cognitive impairment) have been documented in people experiencing modest circadian disruption resulting from jet-lag or a few nights of continuous work [2,9,10]. As early as 2007, the International Agency for Research on Cancer, a branch of the World Health Organization, listed shift work that involves circadian disruption as a “probable carcinogen” (http://www.iarc.fr/). The wide range of behavioral disorders, illnesses, and organ systems affected by disrupting the body’s normal circadian biology suggests that the consequences of long-term shift work are relevant and applicable to providers in many specialties, not just limited to sleep specialists. As a result, there is a sense of urgency in having medical students understand the relative contribution of the environment to diverse aspects of human physiology early in their careers.

## Discussion

### Circadian Rhythms in Medical Education

In medical training programs, research and education regarding sleep/wake disorders, circadian deregulation, and chronobiology is limited to a handful of domain areas including cardiology, otolaryngology, neurology, pulmonology, behavioral disorders, and psychiatry/psychology. These subjects are typically only taught for a short time period and are restricted to a number of specific sleep topics (*e.g.*, sleep apnea, insomnia, sleep disorders) [11]. A recent survey of medical schools across different countries, which had a 20.63% response rate from U.S. and Canadian schools, revealed that American medical schools provide only “*three hours*” of sleep instruction throughout their student’s entire education [11]. Although structured teaching on the topic of circadian-related disorders and chronobiology would be difficult to incorporate in an already demanding curriculum, alternatives include developing core competencies that focus on: *i*) understanding what drives a person’s internal clock, *ii*) the nature of the patient’s circadian disorder (*e.g.*, delayed/advanced sleep phase, irregular sleep/wake, non-24 h (or free-running) sleep/wake, jet lag, shift work sleep), *iii*) how alterations in the circadian system impinge on aspects of human physiology relevant to disease development, and *iv*) how circadian timing can be applied to disease prevention, patient care, and treatment options. The authors propose to integrate these competencies using a longitudinal teaching format within three different levels in the medical curriculum and without the need for developing additional lectures (Table 1).

**Table 1:** Strategies for incorporating circadian rhythm concepts in the medical curriculum.

How/Where to Incorporate Circadian Rhythms Education in Medical School and Beyond

**Medical Students**

**Years 1–2 Curriculum**
– Inclusion of circadian disorders into basic sciences – Develop core competencies: what drives the internal clock?, nature of circadian disorders, relevance to disease development, application to disease prevention, treatment, and patient care – Case-based learning to incorporate circadian disruption-related objectives – Continued training of the medical history to include stress, sleep, and activity levels as part of “Social History” and/or “Review of Systems”
**Years 3–4 Curriculum**
– Inclusion of sleep medicine and circadian rhythms to core rotations (internal medicine, pediatrics, psychiatry, neurology) – Electives in sleep medicine. Opportunities for away rotations at institutions with such a program
**Resident Physicians**

– Addressing circadian rhythms and sleep as it applies to individual specialties – Involvement in research within the circadian/sleep fields (fellowship training program) – Awareness of the effects of sleep deprivation/circadian disruption and their influence on clinical performance; consideration of how these concepts relate to duty hour restrictions
**Fellowship Training**

– Continued support of sleep fellowship opportunities – Incorporate the topic of circadian disruption to a wide range of specialities: family medicine, internal medicine, neurology, psychiatry, pediatrics, otolaryngology – Increasing opportunities for research-based fellowships, particularly to involve translational aspects of sleep medicine
**Attending Physicians**

– Continued application of basic science and clinical knowledge, including the taking of history related to sleep and its disorders – Consider patient social history when evaluating treatment/patient care. Patient education regarding shift work and sleep disorders – Continuing educational opportunites as available

**Figure 1:**
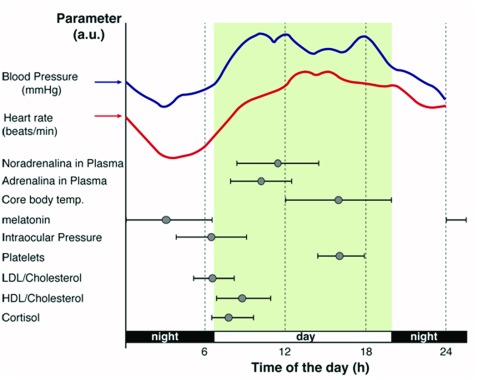
Circadian acrophase chart for various parameters in plasma.

First, we envision an approach in which circadian biology becomes part of the conversation early on in medical school by adding the topic to existing blocks of lectures allocated to, for example, metabolism, endocrine regulation, and gene expression; all of which are processes that have been proven to be linked to circadian rhythms on a molecular level [12,13,14,15]. Application of circadian rhythms to clinical medicine is of relevance as the time of day is an important variable when running medical tests [*e.g.*, when measuring systolic and diastolic blood pressure, intraocular pressure, insulin response, coagulation, and hormonal studies [16,17,18,19], see examples in Figure 1] and most likely influences diagnosis and pharmacotherapy. In addition, conceptualization of biological rhythms in laboratory medicine is certainly relevant to health professionals as it represents a challenge, helps improve diagnostic accuracy, and is an opportunity to better assess the therapeutic efficiency of a given drug. Patient’s samples collected at different times can significantly affect a variety of common diagnostic measurements [20,21] and reference ranges have been defined to make the correct diagnosis of medical conditions and disease states [22].

Because body rhythms influence the pharmacokinetics, pharmacodynamics, and toxicity of therapeutic drugs, there is also a value in incorporating the topic of circadian rhythms within the domain of pharmacology (known as chronopharmacology); specifically, with regard to the timing and dosing at which medications should be administered to either optimize the desired and/or minimize adverse drug effects. Including the concept of chronopharmacology, within the pharmacology curriculum, would provide a contrasting view to the more traditional “*homeostatic*” approach that has become prevalent in medical schools, in which consistency in medication levels are predicted to translate into constancy in drug effects and avoidance of adverse effects. It is certainly clear that concepts such as chronokinetics, dose-time dependent differences in absorption, distribution, metabolism, and elimination of drugs from the body [23,24], and chronodynamics, dose-time dependent differences in drug effects [25], may challenge some long held concepts in pharmacology. Making students aware of the current status of knowledge in these areas will provide them with a more holistic view of what constitutes the best therapeutic strategy.

Specific examples of the application of chronopharmacology and chronotherapeutics in the medical field should be easily incorporated into the curriculum, as reports of giving medications in sync with the patient’s circadian rhythms and their consequences are widespread. Accordingly, circadian variations in blood pressure, heart rate, and other mediators have been shown to increase the risk of stroke and myocardial ischemic events in the morning [26], which led to the examination of cardiovascular medications and the impact of their timing. For example, many studies found a benefit in dosing amlodipine and other cardiovascular medications at bedtime [27,28,29]. Others examined how chronotherapy affects subsets of patients with chronic conditions and risk factors for hypertension or cardiac disease. Remarkably, hypertensive patients with type 2 diabetes that are given medication for hypertension at night exhibited significant decreases in both nocturnal and 24 h systolic blood pressure values [30]. Other prospective studies for chronotherapy regarding antihypertensive medications, demonstrated that bedtime dosing was more effective in controlling the 24 h blood pressure pattern while concurrently reducing adverse effects and cardiovascular disease risk, particularly for those medications targeting the renin-angiotensin-aldosterone system [31,32]. The need for chronocontrol of blood pressure is of relevance to reducing vascular risk associated with chronic kidney diseases, as bedtime ingestion of angiotensin-converting-enzyme inhibitors as well as angiotensin-receptor blockers have proven to be more effective in reducing blood pressure during sleep, as well as, overall than morning intake [33]. Consequently, careful timing of medication dosing provides clinicians with a means to effectively treat hypertension-associated disease severity. Other disparate examples involve the optimization of chrono-based formulation for the treatment of rheumatoid arthritis using low doses of prednisone (clinical trial NCT00650078, [34]) as well as the development of chronopharmacotherapy adjunctive approaches to enhance cognitive-behavioral therapies for treating a resistant form of obsessive-compulsive disorders [35].

Chemotherapy is another area of medicine where including time-of-day-dependent administration of treatments in the form of chronotherapeutic protocols has shown promising results [36]. For example, a recent clinical study established a relationship between circadian rhythms and the variable pharmacokinetics and toxicity of cisplatin for patients with advanced non-small cell lung cancer [37]. Findings revealed that current cisplatin-based chronotherapy protocols show advantages in relieving side effects of chemotherapy in patients by directly influencing the chemo drug’s metabolism [37]. Similarly, chronomodulated chemotherapy of platinum-based drugs and 5-fluorouracil can significantly improve efficacy and reduce adverse events in patients with metastatic colorectal cancer; however, these results must be taken with caution as its effect may be different for the treatment of other solid tumors [38]. Nevertheless, what started as a growing understanding of the importance of biological timing for normal physiology and the pathophysiology of diseases like cancer is now a more comprehensive field that revolves around cancer-associated clock disruptions, clock-dependent mechanisms of cancer, and chronotherapeutic approaches.

Areas of study such as neurology and psychiatry would also benefit from incorporating concepts related to circadian behavior in their lectures, as several lines of evidence show a direct, albeit complex, relationship between sleep and circadian abnormalities and cognitive performance and even neurodegenerative disorders [39,40,41]. Delay, advances, or desynchronizations of circadian rhythms are important pathophysiological factors that influence psychiatric disorders and are, by far, the most widely reported disturbances associated with depression [42]. Changes in daily and seasonal mood variations [43,44,45], brain activity [46,47], core body temperature (*e.g.*, elevation of mean nocturnal body temperature [48]), sleep/wake cycle (*e.g.*, 50 to 90% of depressed patients complain about impairment of sleep quality/duration [47]), and hormone secretion (*e.g.*, cortisol, norepinephrine, prolactin, thyroid stimulating hormone [49,50,51]) are among the most consistent circadian alterations associated with major depression. Because circadian alterations may represent a core component of depression, at least for some patients, they are worth clinical and therapeutic consideration. Today, pharmacological (*e.g.*, melatonin and mood stabilizers alone or in combination) and non-pharmacological (*e.g.*, light therapy and interpersonal and social rhythm therapy) interventions that aim to restore normal endogenous rhythmic patterns are gaining ground as effective strategies to treat various psychiatric disorders. Consequently, programs need to be organized in a manner in which pre-clinical instruction incorporates knowledge about the connections between biological clock functions and behavior. This could be accomplished through individual combinations of lectures, problem-based learning, tutorials, small-group seminars, and even as part of the early contact with patients. Thus, concepts are incorporated at all levels from passive information absorption through didactically-delivered lectures to an active learning system when interacting with patients. We envision that these strategies would help health professionals in identifying feasible and non-conventional treatments for resetting the body’s rhythm and could be used as either alternative or adjunct therapies to treat behavioral disorders.

Second, since almost every physiological variable that is taken into consideration as a diagnostic indicator in the clinic has been shown to exhibit circadian rhythmicity [52], it should be fairly straightforward to incorporate concepts related to circadian rhythms as additional material in patient clinical case discussions, particularly during the first two years of medical school. In fact, medical students are already instructed to include questions regarding occupation, sleep/wake habits, and stress within the social history portion of the patient interview. Thus, additions regarding a patient’s sleep disruption history should not be daunting. With all this information on hand, students would be expected to develop hypotheses and make decisions that incorporate aspects of the patient’s environment, thus, weighing the risk that an alternative shift work history for a given patient may represent and considering its influence on therapeutic treatment.

Clinical rotations during the last two years of medical school present additional opportunities for the introduction of circadian knowledge. Core disciplines may incorporate the subject topic in a variety of formats; for example, pediatric medicine can take into consideration concepts that relate to circadian markers in pediatric obstructive sleep apnea, circadian-linked metabolic deregulation in early childhood, changes in rhythms during puberty, the clinical use of melatonin in pediatrics, along with topics linked to sleep disordered breathing and bipolar disorder in infants [53,54,55]. Furthermore, the clinical discipline of internal medicine provides a unique scenario for developing concepts in, for example, the circadian-immune connection and in cardiovascular health and disease prevention in a system whose functional organization has clear links to circadian rhythmicity [56,57]. Moreover, the body’s chronodisruption directly affects, among others, gut motility, gastric acid secretion, maintenance and restoration of the mucosal barrier, production of digestive enzymes, and nutrient transport in the small intestine, therefore influencing the development of inflammatory and functional gastrointestinal diseases including bowel disorders, non-alcoholic hepatic steatosis, and even some types of cancers (for review see [58]). Rotations in obstetrics/gynecology present a great opportunity for discussing the relationship of, for example, uterine circadian activity with term and preterm delivery [59], the pulsatile nature of luteinizing hormone and testosterone secretion [60], and the day/night rhythms of endocrine organs [61].

Lastly, elective rotations, often placed predominantly during the fourth year in the medical curriculum structure, may provide an excellent opportunity for developing the subject area of circadian rhythms and associated diseases, particularly if offered by institutions that have affiliated sleep centers. These electives would allow students additional exposure to the specialty and additional instruction, particularly for students that do not anticipate completing a sleep fellowship but may desire additional knowledge for their future practice. The flexibility that many medical schools have regarding travel for elective rotations would also allow fourth year students from schools that do not have sleep centers to travel to schools that do. In these ways, the last two years of medical school present a unique opportunity for students to develop these skills before entering a diverse range of specialties.

## Beyond the Classroom Curriculum

The recommendations to the medical school curriculum summarized in Table 1 would likely fall within the fellowship training programs rather than influencing changes at the residency level, at least, in the short term. One of the early approaches to introduce the concept of circadian disruption and its physiological implications was led by Stanford University through the creation of the Accreditation Council for Graduate Medical Education (ACGME) sleep disorder medicine fellowship training program at the Stanford Center for Sleep Sciences and Medicine (http://stanfordhospital.org/clinicsmedServices/clinics/sleep/). Here, professionals are trained in multiple aspects of sleep medicine including pharmacology of sleep, disordered breathing, neuro-degenerative disorders, insomnia, narcolepsy, and orthodontics in children and adults, among other relevant topics. Although the aforementioned program, and other outstanding fellowship programs taught at various Sleep Centers nationwide, focuses on one aspect of circadian deregulation, sleep, their efforts constitute a valuable first step towards understanding and strategically incorporating specialized topics related to rhythms at the upper level of education. A long-term goal might include the establishment of an alternate research-based fellowship program to promote career development of clinician researchers in the area of circadian disorders that would consider incorporating research aspects of circadian biology into the clinical practice, a relevant topic to both ACGME and the Center for Sleep and Wake Disorders, National Institutes of Health. Finally, physical, mental, and behavioral changes associated with alterations in the organism’s environment are topics of clinical and research relevance that can be examined within any of the residencies (family medicine, internal medicine, neurology, psychiatry, pediatrics, and otolaryngology) required for a medical sleep disorders clinical fellowship. Creating a residency area that specifically advocates for gene-environment interaction and their deregulation in various diseases and for treatment would likely take time to implement but would be worth pursuing in the long-term.

Lastly, an additional topic that deserves to be mentioned for further discussion, although it is beyond the scope of the present article, refers to disruptions that the hospital environment itself poses to medical students, nurses, physicians, technical personnel, and patients as they are exposed to relatively constant levels of noise, light, and activity around the clock, especially in intensive-care units and in patients that have remained admitted to the hospital for several days. Although a subject of considerable controversy in both Europe and America, there have been steps already taken to evaluate, set, and enforce standards for effective medical residency that considers circadian disruption as a factor that influences occupational stress, fatigue, and, ultimately, performance in medical residents and hospital personnel in general [62,63,64,65,66].

## Enhancing a Safe Circadian Environment in a Clinical Setting

Circadian rhythm deregulation is a “*two-way street*” when considering its importance in medical residency and fellowship training programs. Not only does it represent the last opportunity to address the relevance of circadian rhythms in disease onset and progression to health professionals in their final stage of training but it also is a topic that affects medical students as they are among the professionals that are most heavily exposed to circadian disruption during their training years.

The intention of this article is not to formulate programmatic changes or discuss the effectiveness and extent to which the 80 h per week work schedule has been implemented, or not, in medical residencies; instead, it is meant to bring to the reader’s attention the problems associated with circadian disruption that affect the health and performance of providers in a clinical setting. In a landmark study, Landrigan *et al.* brought to our attention the need for eliminating extended work shifts and implementing a weekly hours cap for health care providers in order to reduce serious medical errors attributed to impaired neurobehavioral performance due to sleep deprivation [67]. Of note is that, whereas the Landrigan *et al.* study might have been methodologically more rigorous than previous work on the subject, reports that date back decades describe how circadian disruption, in the form of sleep deprivation, compromises neurocognitive response, physicians’ clinical performance, and patients’ safety [68,69,70,71,72,73,74,75]. As a result, these studies expose the level at which acute and chronic partial sleep deprivation influences errors in intervention, medication, and diagnosis and urges the health community to “*mitigate the deterioration in performance resulting from circadian misalignment* [67]” by reforming the provider’s work schedule; a suggestion that has been heard by the ACGME and that resulted in a number of recommendations to which hospitals need to adhere but are not necessarily enforced (ACGME, 2011; http://www.acgme.org/acgmeweb/Portals/0/PDFs/dh-faqs2011.pdf).

Thus, there is value in emphasizing the many physiological consequences, other than cognitive, that circadian disruption might also have on providers and the long-term impact of these consequences on their health and that of their patients. It is not about knowing the potential impact of circadian disruption in diagnosis and treatment of patient’s diseases but, also, acknowledging the potential impact to those that bear the decision-making role and heavy responsibility in the clinic.

## Conclusions

Since socio-economic factors influence the distribution of a growing work force of individuals who maintain schedules that are in direct conflict with their body’s physiology, the contribution of environmental factors to disease development, progression, and treatment are increasingly relevant. As such, there is an urgent need to make medical professionals fully aware of the multiple gene-environment interactions that influence patients’ health and their response to therapies. Thus, including circadian-related topics in the medical curriculum will facilitate transition from a reductionist approach to disease understanding to consider a more holistic framework in which the environmental context in which the individual lives becomes part of the disease’s evaluation.

## Competing interests

The authors declare that they have no competing interests.
